# Ribosome specialization in cancer: a spotlight on ribosomal proteins

**DOI:** 10.1093/narcan/zcae029

**Published:** 2024-07-09

**Authors:** Sofia Ramalho, Anna Dopler, William James Faller

**Affiliations:** Division of Oncogenomics, The Netherlands Cancer Institute, Amsterdam, Netherlands; Division of Oncogenomics, The Netherlands Cancer Institute, Amsterdam, Netherlands; Division of Oncogenomics, The Netherlands Cancer Institute, Amsterdam, Netherlands

## Abstract

In the past few decades, our view of ribosomes has changed substantially. Rather than passive machines without significant variability, it is now acknowledged that they are heterogeneous, and have direct regulatory capacity. This ‘ribosome heterogeneity’ comes in many flavors, including in both the RNA and protein components of ribosomes, so there are many paths through which ribosome specialization could arise. It is easy to imagine that specialized ribosomes could have wide physiological roles, through the translation of specific mRNA populations, and there is now evidence for this in several contexts. Translation is highly dysregulated in cancer, needed to support oncogenic phenotypes and to overcome cellular stress. However, the role of ribosome specialization in this is not clear. In this review we focus on specialized ribosomes in cancer. Specifically, we assess the impact that post-translational modifications and differential ribosome incorporation of ribosomal proteins (RPs) have in this disease. We focus on studies that have shown a ribosome-mediated change in translation of specific mRNA populations, and hypothesize how such a process could be driving other phenotypes. We review the impact of RP-mediated heterogeneity in both intrinsic and extrinsic oncogenic processes, and consider how this knowledge could be leveraged to benefit patients.

## Graphical Abstract

**Graphical Abstract:**
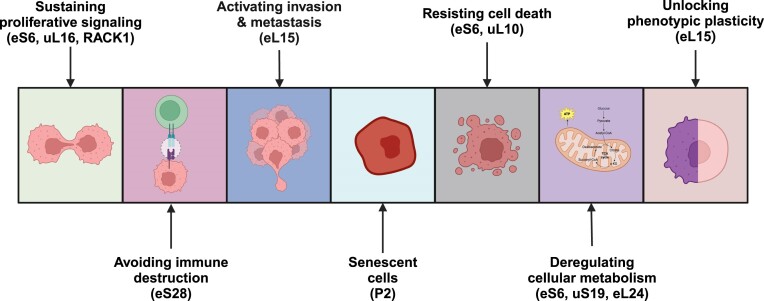

## Introduction

Since the establishment of the central dogma of molecular biology in 1958, very few cellular components have been studied as intensively as the ribosome (1). This work has spawned thousands of papers, and two separate Nobel prizes (2). As a result, it is not particularly surprising that for decades, there was a general view that we understood the ribosome: that it was a passive molecular machine with no inherent regulatory capacity. Furthermore, the prevailing view was that, outside of a small number of exceptions, ribosomes were a homogeneous population.

However, science is a constantly surprising endeavor, and, beginning in the 1980s, cracks began to appear in this assumption. Over the last number of decades, these cracks have gotten wider and wider, and it is now accepted that there is substantial heterogeneity to the ribosome. This heterogeneity comes in many flavors, including in both its RNA and protein components. The last decade in particular has brought many groundbreaking insights. The identification of ribosomes that translate specific pools of mRNA (3), and the recent description of the remodeling of eukaryotic ribosomes in the cytoplasm (4–6) which built on previous work showing the same phenomenon in bacteria (7,8), suggests a far more dynamic molecular complex than has previously been appreciated. While we are still at the genesis of this field, we are now beginning to understand some of the basic concepts that underlie it, and how it is co-opted in disease.

The earliest reported relationship between RPs and cancer was published in the 1990s, which demonstrated the association of eS19 (RPS19) expression levels with colon carcinoma progression and differentiation (9). However, this is one of a large number of studies that correlate RP expression with outcomes (10), not differentiating between ribosomal and extra-ribosomal function of an RP (which have been extensively described elsewhere (11)). This differentiation is still a challenge today (see the Technical challenges section below). uL5, for example, is one of several RPs known to interact with MDM2, thus acting as a regulator of p53 (12), and such extra-ribosomal functions can be hard to separate from a specific role in ribosome specialization. A key open question is how much of the observed heterogeneity in ribosomes translates to a specialized function.

This has been a longstanding source of discussion, as aside from extra-ribosomal functions, changes in RP levels in ribosomes could simply change the availability of functional ribosomes (the ribosome concentration hypothesis) (13). It is known that several ribosomopathies show little or no difference in ribosome composition, suggesting that overall ribosome levels are key in these cases (14,15). While this debate is still ongoing (and has been reviewed elsewhere (13,16)), there are now several studies that show distinct functions for ribosomes with divergent RP complements, indicating a place for both the ribosome concentration hypothesis and the ribosome specialization hypothesis (3,17–19). Development and refinement of methodologies will allow us to get a much clearer idea of the biological contexts that rely on each of these forms of ribosome alterations.

At present, half of the established Hallmarks of Cancer (20) have been shown to be regulated via ribosome specialization in some contexts (summarized in the Graphical Abstract), and this is likely to increase in the coming years. In this review, we will focus on the evidence for a role of specialized ribosome populations in these processes. In particular, we will highlight RP-mediated ribosome specialization focusing on either changes in RP stoichiometry, or RP post-translational modifications (PTMs). As we have described above, changes in RP levels do not necessarily indicate ribosome specialization, so we will concentrate only on examples that show specialized translation programs, or describe modifications whose functional consequences rely on ribosome function. While there is a limited number of studies currently available (summarized in Table 1), they strongly indicate a role for ribosome specialization in cancer.

**Table 1.** RPs shown to regulate cancer phenotypes via ribosome specialization. Only RPs known to impact the translation of specific panels of mRNAs are included

RP	Modification	Translational program	Tumor type
RACK1	Incorporation (50,55)	Translation of mRNAs to promote survival	Neuroblastoma, HCC
eS6	Phosphorylation (34,36–39,101), MARylation (83)	TOP-motif & HIF-1ɑ translation, polysome assembly	DLBCL, breast cancer, cervical carcinoma, ovarian cancer
uS19	Incorporation (96)	Cap-independent translation and stop codon readthrough	CLL
eS28	Incorporation (145)	Non-canonical translation of DRiPs for immune surveillance by CD8 + T cells	Melanoma
uL10	Incorporation (129)	Translation of survival regulators (via eIF2ɑ phosphorylation)	Leukemia
eL15	Incorporation (134)	Translational of RP-encoding mRNAs to promote metastasis	Breast cancer
uL16	Mutation (43,97,98)	Translation of mRNAs to promote survival via JAK/STAT signaling; IRES-mediated translation	T-ALL
eL24	Acetylation (57), Mutation (60), MARylation (83)	eIF4E-dependent translation, polysome assembly	Breast cancer, CRC, ovarian cancer
P2	Incorporation (131)	Translation of senescence-associated mRNAs	Fibrosarcoma

## RPs in oncogenic signaling

**Figure 1.**
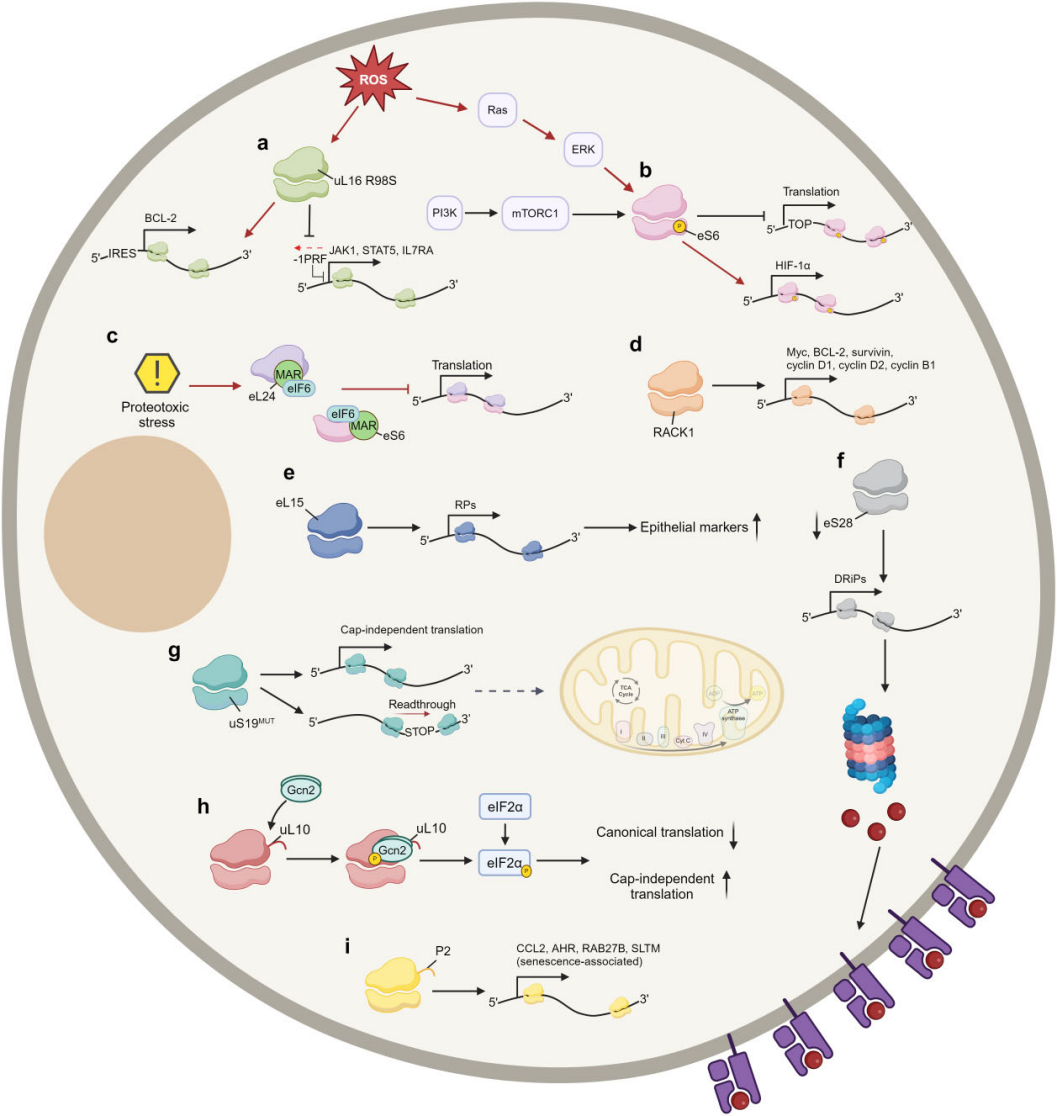
Signaling pathways and phenotypes regulated by ribosome specialization, and which RP mediates this regulation. (**a**) Upon accumulation of ROS, ribosomes containing mutated uL16 R98S promote IRES-mediated translation of BCL-2. These ribosomes are also involved in the upregulation of JAK-STAT family members, through inhibition of programed −1 ribosomal frameshifting (-1PRF). (**b**) PI3K/AKT/mTORC1 signaling axis promotes phosphorylation of ribosomal eS6 regulating the translation of TOP-motif containing mRNAs. ROS-induced ribosomal phospho-eS6 leads to an upregulation of translation of HIF-1ɑ. (**c**) Proteotoxic stress induces MARylation of eL24 and eS6 resulting in increased ribosome association of elF6, inhibited 80S assembly and reduced translation of proliferative genes. (**d**) RACK1 promotes translation of mRNAs encoding for growth and survival factors, such as Myc, BCL-2, surviving, cyclin D1, cyclin D2 and cyclin B1. (**e**) eL15-containing ribosomes promote metastasis by preferential translation of RP-encoding mRNAs required for increasing epithelial markers’ expression. (**f**) Depletion of eS28 on the ribosome increases total peptide presentation via the production of defective ribosomal products (DRiPs), and enhances tumor visibility and killing by T cells. (**g**) Ribosomes containing mutated uS19 promote cap-independent translation and stop codon readthrough to downregulate mitochondria-associated pathways such as pyruvate metabolism, TCA cycle, and respiratory electron transport. (**h**) Interaction of Gcn2 with uL10-containing ribosomes results in Gcn2-dependent phosphorylation of eIF2ɑ and consequent downregulation of canonical translation and upregulation Cap-independent translation to promote tumor survival. (**i**) P2-containing ribosomes regulate translation of mRNAs associated with tumor senescence, such as CCL2, AHR, RAB27B and SLTM. Figure created with Biorender.com.

The capacity for sustained limitless proliferation and the ability to evade cell death were some of the first described hallmarks of cancer (21), and are at the foundation of tumor development. Numerous signaling pathways are rewired to potentiate growth and survival, and the role of genetic alterations in this process has been extensively described (22–24). However, as a bridge between the genome and proteome, RNA translation also represents a crucial layer of signaling regulation in cancer cells (25,26). Many of the signaling networks hijacked by tumors regulate translation, and mRNAs involved in almost every aspect of cancer have been reported to be regulated by this process (27,28). However, the role of ribosome specialization in oncogenic translation is still largely unknown, although a number of convincing examples have been published (summarized in Figure 1).

### mTOR signaling

Due to its association with the PI3K/AKT/mTORC1 signaling axis, ribosomal protein eS6 (RPS6) is the most well-known example of an RP involved in oncogenic signaling. mTORC1 is a direct regulator of translation at many levels (29–31), and eS6 was one of its first identified targets (32). Indeed, phosphorylation of eS6 is often used as a proxy for mTORC1 activation. Despite this, the function of this PTM has still not been fully elucidated, although it has been described to have a wide impact on translation (33).

eS6 has been shown to be specifically associated with mRNAs containing 5’-terminal oligopyrimidine tract (TOP) motifs, inhibiting translation of these transcripts. Knock-down of eS6 leads to an upregulation of TOP mRNA translation in primary diffuse large B-cell lymphoma (DLBCL), breast cancer cells (MCF7) and cervical carcinoma cells (HeLa) (34,35). These studies did not look at the role of eS6 PTM, however rapalog treatment (which inhibits mTOR-mediated eS6 phosphorylation) has shown that phosphorylated eS6 promotes TOP mRNA translation (35–37), suggesting that eS6 phosphorylation and eS6 down-regulation phenocopy each other. As RPs themselves are commonly regulated via TOP motifs, this results in a complicated feedback loop. Particularly, the eS6-mediated regulation of uL5 (RPL11) provides an interesting example. As mentioned above, uL5 has an extra-ribosomal role in p53 signaling. eS6 promotes the translation of uL5, thus promoting p53 function (35,38), and significantly affecting cellular phenotypes (12).

However, the role of phosphorylated eS6 in translating uL5 and other TOP-mRNAs has more recently been challenged. A 2021 study reported that its role in translation depends on the coding sequence (CDS) length, promoting efficient translation of mRNAs with short CDSs (39). Even though TOP-motif containing mRNAs are known to have short CDSs, this study suggests that they represent an exception, being resistant to loss of eS6 phosphorylation in mouse embryonic fibroblasts (MEFs). The authors proposed that translation of TOP-mRNAs may be influenced by an eS6 phosphorylation-independent mechanism. Although it is clear that eS6 phosphorylation plays a multilayered role in translation, production of proteins from TOP-motif containing mRNAs appears to be a major function of eS6-containing ribosomes in some contexts, and both native and modified forms of this RP appear to be associated to specific translational programs.

### Jak-Stat signaling

Another cancer-related signaling pathway that is known to be affected by ribosomal alterations is the Jak-Stat pathway, which regulates embryonic development and other processes, such as stem cell maintenance and inflammatory response (40,41). The R98S mutation in *Rpl10 (*uL16), which is present in 7.9% of pediatric T-cell acute lymphoblastic leukemia (T-ALL) cases, has been shown to influence Jak-Stat signaling (42). This ribosome modification specifically increases protein levels of members of the Jak-Stat pathway by altering their programmed −1 ribosomal frameshifting (-1 PRF) rates. This leads to an uncontrolled activation of this oncogenic pathway (43,44), although the precise mechanism through which this mutation increases −1 PRF is not known. However, its prevalence in T-ALL and its clear role in oncogenic signaling, led the authors to describe ribosomes containing this mutated form of uL16 as ‘onco-ribosomes’ (45), although it is likely just one example of specialized ribosomes supporting oncogenic phenotypes (46).

### Myc signaling

Myc signaling is well known to regulate translation, primarily through its influence on ribosome biogenesis, via the promotion of the transcription of mRNA precursors of ribosomal components, such as RPs (47). Conversely, multiple RPs have also been shown to impinge on Myc signaling, both as up-stream and down-stream regulators of Myc.

One example is the receptor for activated C kinase 1 (RACK1). Primarily described as a scaffold protein for protein kinase C (PKC), RACK1 has a wide interactome, serving a scaffolding function for many proteins and thereby playing a role in various processes such as cell migration, viral infection and angiogenesis (48,49). The role of RACK1 expanded with its classification as an RP, which functions as a signaling hub on the ribosome through recruitment of kinases and mRNA-binding proteins (RBPs) (49,50). In addition to having an impact on overall translation (51,52), RACK1 is not essential in cells (53) and exhibits a dynamic association with ribosomes, suggesting a possible involvement in ribosome heterogeneity (53,54). In fact, this RP mediates specific oncogenic translation programs, modulating the translation of Myc and other mRNAs that encode for growth and survival factors (such as survivin and BCL-2) (50), and cell cycle regulators (such as Cyclin D1, D3 and B1) (55), in both neuroblastoma and hepatocellular carcinoma (HCC), respectively.

eL24 (RPL24) has also been described to play an important function in ribosome assembly following Myc activation (56,57). It has also been shown to support the *in vivo* oncodriver potential of both Myc and Akt, independently of any impact on overall ribosome assembly (58,59). In the context of breast cancer, depletion or inhibition of this protein, via acetylation, impairs eIF4E-dependent translation, affecting the expression of proteins such as cyclin D1, survivin and NBS1 (57). In colorectal cancer (CRC), reduced levels of eL24 in the *Rpl24^Bst^* mouse suppress tumorigenesis in an eEF2-dependent manner, with no changes in overall ribosomal subunit abundance (60), a mechanism that is also seen in melanocytes (61).

### Wnt signaling

A recent study has shown that uL1 (RPL10A)-containing ribosomes are important for translation of Wnt pathway mRNAs in embryonic development (18). Wnt signaling is a well-established oncogenic pathway. It plays a driver-role in several cancer types, such as colorectal cancer, mediating uncontrolled proliferation, stem cell maintenance, metastasis and immune response (62). Even though the role of uL1-containing ribosomes in cancer development has not been explored yet, it will be interesting to understand whether this specialized ribosome population could have the same impact in Wnt-driven cancer as it has in development.

### Hox signaling

Another RP that has been described to be a mediator of ribosome heterogeneity in the context of development is eL38 (RPL38). A pair of studies by the Barna lab has shown that eL38-containing ribosomes specifically regulate the translation of Hox genes, through recognition of elements in their 5’UTRs (19,63). Some of the Hox genes described as being translationally controlled by these ribosomes, such as *Hoxa5* and *Hoxa9*, have a well-established role in oncogenic processes, such as proliferation, survival and differentiation, in several cancer types (64,65). For example, HOXA9 has been established as a driver of proliferation in acute myeloid leukemia (AML) and its therapeutic value in this disease has been explored in a number of studies (64). Currently, the role of eL38-containing ribosomes in cancer development is only correlational; however, it would be interesting to explore whether this specialized ribosome population is an upstream regulator of oncogenic Hox signaling.

### Other signaling pathways

eL29 (RPL29) is a less direct example of an RP associated with oncogenic signaling. This RP is not essential to the ribosome but plays an important role in translation efficiency (66), highlighting it as a potential heterogeneously incorporated component of ribosomes. eL29 has been identified as a major substrate for methylation by Set7/9 (also known as Set7, Set9, Setd7 or Kmt7) (67), which is a modulator of the Hippo/YAP and Wnt pathways in many cancers (68,69). This modification was shown to increase nuclear localization of eL29, with no effect on global protein synthesis. These findings hint at the possibility of the existence of ribosomes lacking eL29, and this process being regulated by Set7/9. However, changes in incorporation of this RP upon methylation, the functional relevance of this finding, its impact on the translation of specific mRNAs, and its role in cancer models still need to be assessed.

Two RPs that have also been described to be involved in specialized translation are eL36 (RPL36) and eL42 (RPL36A). Ribosomes containing these RPs appear to regulate the translation of Hsp90, which is implicated in many aggressive tumor types, and acts as a chaperone for many oncogenes (70–73). However, this last example is a more correlative one, and further mechanistic explanation of this regulation is needed.

Although the examples given here all relate to the regulation of oncogenic signaling, the reverse may be true, and these signals themselves may also regulate the abundance of specific ribosome populations. Myc, for example, is a well-described regulator of ribosome biogenesis, and it is easy to imagine that it can regulate the production of various ribosome populations via the differential transcription of ribosomal components. Although at present there is no evidence of this occurring, it is an obvious area of future research.

## RPs in stress responses

Due to their high growth rate, their often nutrient-depleted environment, and their metabolic demands, cancer cells are constantly exposed to stress. Add to this the systemic attempts to eliminate them, clonal competition, and therapy exposure, and it is clear that cancer cells must exist in a state that allows them to overcome all these hurdles. As a result, cancer cells activate stress responses, which allow cells to survive, proliferate and resist therapy (74–76). During stress, protein synthesis is often down-regulated; however, this is often accompanied by a rewiring of overall translation, with increased translation of selected transcripts that aid in survival and recovery. Several mechanisms have been shown to allow such specific translation, and there are indications that ribosome specialization may be one such layer of regulation (27,77). Clearly there is a high level of cross-talk between various stress responses; however, for the sake of simplicity, we have discussed each in turn below.

### Proteotoxic stress

Cell growth is obviously required for cell division and proliferation, and the levels of protein synthesis must be kept within certain bounds to ensure the health of the cell, a process known as proteostasis (78). Cancer cells are often exposed to proteotoxic stress (79,80), and ribosomes are central to their response to this, something that is largely mediated by PTMs of RPs (81,82). One example is the increased MARylation (addition of mono (ADP-ribosyl)) of eL24 (RPL24) and eS6 (RPS6) following proteotoxic stress. This leads to an increased association of eIF6 with ribosomes, inhibiting 80S assembly, and decreasing proteotoxic stress as a result (83). Interestingly, acetylation of eL24 can also be anti-tumorigenic, by reducing the translation of proliferative genes and inhibiting assembly of the 80S (57). In this case, impairment of translation by modified eL24 is deleterious for proliferation of cancer cells, suggesting that a balance in the levels of eL24 modification is needed for proper proliferation. This allows for increased translation to sustain proliferation with a negative feedback loop to avoid proteotoxic stress and consequent cell death. The type of eL24 modification can play a role in this fine-tuning as well as the combined modification of other RPs; however, these hypotheses still need to be investigated.

A key component of the proteotoxic stress response is the unfolded protein response (UPR), activated as a consequence of the accumulation of misfolded proteins in the endoplasmic reticulum (ER) (76,79,84). UPR is a process that is active in many cancer types and supports oncogenesis and chemoresistance (85). There have been some RP PTMs identified to take place upon UPR, such as ubiquitination of uS5 (RPS2) and uS10 (RPS20). These modifications of the 40S communicate proteotoxicity to the translation machinery, and are essential for preventing UPR-induced cell death (86). However, the role of these RP modifications in UPR still needs to be further understood and additional experiments on their role in the context of cancer need to be performed. There is a strong chance that this ubiquitination can mediate translation rewiring, similar to what has been seen in UPR-mediated ubiquitination of eS7 (RPS7) in yeast (87). However, this has not yet been shown in mammalian cells, so whether it contributes to a functional ribosome specialization in cancer is not known.

### Metabolic stress

Another common stressor that cancer cells can experience is metabolic stress, which comprises several aspects such as nutrient starvation, hypoxia and oxidative stress with accumulation of reactive oxygen species (ROS), all of which result from the fast uncontrolled growth of tumors, and ultimately lead to processes such as apoptosis or autophagy (88–90). It has been proposed that modifications of ribosome-associated RPs can play a role in these processes, a clear example of which comes from yeast, where it has been shown that RPs can act as oxidative stress sensors (91,92).

To face the high demand for energy that a high proliferative state requires, the metabolism of cancer cells is highly optimized (93). The aforementioned R98S mutation in uL16 results in the overexpression of several metabolism-related proteins (43); however, this observation is not further explored. In chronic lymphoblastic leukemia (CLL), mutated uS19 (RPS15) (94,95) is incorporated into ribosomes, and this was shown to promote cap-independent translation and stop codon read-through. These cells acquire proteomic changes, downregulating mitochondria-associated pathways such as pyruvate metabolism, TCA cycle, and respiratory electron transport chain. Although ribosome profiling is needed, this hints at mutant uS19 changing the translational profile of cells, resulting in the reprogramming of metabolism (96).

Despite changes in metabolism that often accompany oncogenic transformation, oxidative stress is an extremely common event in cancer. The strongest evidence of the role of RP-mediated ribosomal heterogeneity in coping with such stress comes from T-ALL-associated ribosomes containing mutated uL16 R98S (discussed above). Aside from its impact on Jak-Stat signaling, it has been shown that, although this mutation leads to an accumulation of ROS and consequent impaired cell proliferation, it also makes cells resistant to this stress, preventing their death. The specificity of uL16 R98S-containing ribosomes towards IRES-mediated translation of BCL-2 is behind this survival mechanism (45,97,98). It would be interesting to explore whether resistance to oxidative stress driven by this pool of ribosomes is also present in other cancers as well as evaluate whether acquisition of mutations in *Rpl10* could be a possible resistance approach adopted by cancer cells during tumor development.

Besides its established roles in tumorigenic signaling, phosphorylation of eS6 has also been described to modulate signaling related to oxidative stress resistance. Low doses of arsenic were shown to induce expression of the hypoxia-inducible factor-1ɑ (HIF-1ɑ) and consequently trigger angiogenesis to cope with arsenic-induced ROS accumulation (99,100). Interestingly, this arsenic-associated HIF-1ɑ expression is translationally regulated by Ras/Raf/MEK/ERK-activated phospho-eS6 (101).

Oxidative stress plays a role in both tumor initiation, and when solid tumors reach a size that impairs their total vascularization, leading to hypoxia (88). Under hypoxic conditions, eS12 (RPS12) is less present in polysomes, and enriched in monosomes in HEK293T cells (102), which results in increased translation of some structured mRNAs. The same study also demonstrated the existence of spliced isoforms of RPs that are differentially incorporated into ribosomes in hypoxic conditions (102). One of these is eS24, whose splicing has been associated with tumor incidence (103–105). These data suggest extensive alterations to the ribosome under hypoxic conditions, something that deserves to be explored further in an oncogenic context.

### Translation stress

As mentioned above, the high biosynthetic requirements of cancer cells has another consequence: nutrient deprivation and a resulting translation stress (106–109). Several pathways are involved in counteracting this, such as the ribotoxic stress response (RSR), ribosome quality control (RQC) and the integrated stress response (ISR) (110,111). Moreover, the phenotypic consequences of signaling via these pathways share many similarities with cancer-related features, such as cell apoptosis/survival control (112), metabolic regulation (113), cell identity plasticity (114) and inflammatory response (115,116). These stress responses have also been shown to mediate therapy outcomes in different cancer cells (117–119).

Modifications of ribosome-associated RPs have also been found to be at the center of the response to ribosomal impairment. RQC has been shown to trigger ubiquitination of eS10 (RPS10) and uS10 (RPS20) via ZNF598 (120,121), uS5 (RPS2) and uS3 (RPS3) via ribosomal protein RACK1 (121) and, upon initiation of RQC, via RNF10 (122). These PTMs seem to be necessary for resolving ribosomal stress; however, the mechanism used for this resolution, whether by mechanical and conformation changes of the ribosome or by changes in the cell's translation program, is not understood yet. Even though connections between cancer development and ribosomal stress pathways need further exploration and there is no known direct contribution of RP-mediated ribosome specialization in the regulation of these pathways (123,124), the crucial role that RP modifications seem to play in RQC leaves open a possible role in modulating translation stress response in cancer.

## RPs in proliferation arrest, resistance to cell death and senescence

A strategic proliferation arrest of tumor cells is critical for tumor dormancy, delayed disease recurrence, and therapy resistance. Two different types of proliferation arrest can lead to this dormancy state of tumor cells: quiescence, defined by a reversible cell cycle exit to G0; and senescence, defined by an irreversible arrest in G1 or G2, increased senescence-associated β-galactosidase (SA-β-Gal) activity, chromatin changes and the senescence-associated secretory phenotype (SASP) (125).

The terms ‘quiescence’ and ‘dormancy’ are often interchangeable in cancer, and this state is often triggered by exposure to stress conditions (125,126). It is known that quiescent cells show resistance to chemotherapy, and therefore this process can have a very significant impact on disease outcome for patients (127). FXR1 is elevated in G0 and chemosurviving acute monocytic leukemia (AML) cells, and this resistance-phenotype seems to be driven by a variety of ribosomal alterations, one of them being an increase in the incorporation of the ribosome stalk protein uL10 (RPLP0). This is followed by an increased interaction of Gcn2 with the ribosome, and consequent activation of its kinase activity. These ribosomal changes lead to the phosphorylation of eIF2ɑ, which modulates survival by inhibiting canonical translation, and favoring translation of specific survival genes, which are usually translated by non-canonical mechanisms (128). Most importantly it seems that this eIF2ɑ phosphorylation and consequent translatome changes contribute to chemosurvival (129).

When it comes to senescence, this form of proliferation arrest can have both an anti- and pro-tumorigenic role. Even though this aging-associated process is linked to a reduction of protein synthesis, studies have shown that there is still production of proteins needed for the maintenance of this stress-induced phenotype (130). It has been reported that the ribosomal P-stalk, more specifically P2 (RPLP2)-containing ribosomes, are responsible for mediating expression of some of the elements of this senescence-associated translatome (131). Aside from this description of the senescent ribosome, evidence for the existence of a senescence-associated subpopulation of ribosomes is scarce.

## RPs in metastasis

Despite it being responsible for most cancer-related deaths, the process of metastasis remains poorly understood (132). One known driver is epithelial-to-mesenchymal transition (EMT), a developmental program that enhances mobility and invasion of tumor cells, thereby boosting their metastatic potential. Once metastatic cells have arrived in a suitable secondary site, they undergo mesenchymal-to-epithelial transition (MET), a final step in metastasis, which aids in the colonization of this new site, and growth of the metastatic tumor (133).

While most published RP-related studies focus on their extra-ribosomal functions during metastasis development, recent evidence also suggests a role of translational control in this context. Ebright *et al.* reported that altered translation by eL15 (RPL15)-containing ribosomes promotes breast cancer metastasis. Considering that only a small subset of circulating tumor cells (CTCs) form metastatic lesions, the authors used a genome-wide CRISPR activation screen in breast cancer-derived CTCs to identify eL15 as a gene that promotes metastasis formation. Ribosome profiling of eL15-enriched versus control CTCs revealed the preferential translation of virtually all RP-encoding mRNAs, and other translational regulators, by eL15-containing ribosomes. Furthermore, eL15 over-expression enhanced metastatic growth in distant organs. The authors suggest that upon MET, there is a need for increased RP production, which is mediated by eL15-containing ribosomes, highlighting the key role that ribosome specialization plays in this process (134).

It is important to note that the majority of the published work does not provide direct evidence for ribosome heterogeneity in tumor metastasis, and rather look at overall RP levels. However, these findings provide valuable insights into the role of RPs in this context and could serve as a starting point to investigate the potential of specialized ribosomes in this field. As many of these studies look at overall RP levels, looking directly at RP incorporation, for example, could be extremely informative.

## RPs in immune evasion

The ability of cancer cells to avoid immune destruction is one of the core hallmarks of cancer (20). Immune evasion by tumors can have many causes (135), including changes to the peptide repertoire presented on major histocompatibility complex class I molecules (MHC-I), reduced visibility by downregulation of antigen processing and presentation (APP) machinery components (136–138) and the absence of T cell attracting chemokines such as CXCL9 and CXCL10 (139).

A prerequisite for antigen presentation is the translation of source proteins by ribosomes, and the major source of peptides are structurally stable, retired proteins (retirees). While these proteins exhibit rather slow turnover kinetics (median half-life of ∼46h across the entire proteome (140)), immune responses are extremely rapid, and CD8^+^ T cells can recognize aberrant cells within 60 minutes (141). This paradox is explained by the existence of defective ribosomal products (DRiPs) which are translational products that do not achieve stable protein structures and are therefore rapidly degraded and presented on the cell surface (142,143).

The close association between peptide generation by the ribosome and immune surveillance suggests an underappreciated role of ribosomes to build efficient immune responses. A specialized ribosome population that couples protein synthesis to antigen presentation would make sense from an evolutionary point of view, and would at least partially explain the rapid presentation of peptides to CD8^+^ T cells. These ‘immunoribosomes’ are a hypothesized population of specialized ribosomes that preferentially translate antigenic peptides for immune surveillance (144), and the stoichiometry of RPs has been of particular interest in this context.

Wei et al. provided the first clear evidence for the existence of such ‘immunoribosomes’, and to date, this is the only clear published example of ribosome populations playing a role in immune evasion. In this key study, the authors identified a set of RPs that regulate MHC-I peptide presentation for immune surveillance (145). By using an shRNA approach, they identified three RPs, eL28 (RPL28), eL6 (RPL6) and eS28 (RPS28), with pronounced effects on peptide generation or MHC-I levels, without affecting overall translation. While the loss of eL28 resulted in the increase of ubiquitin-dependent and -independent peptide presentation, depletion of eS28 increased total peptide presentation, via the production of DRiPs. Loss of eL6, on the other hand, had the opposite effect, decreasing total peptide presentation. Functionally, in melanoma cells, knock-down of eS28 resulted in increased killing by T cells in a co-culture assay, consistent with increased peptide presentation (145).

In addition to these examples, it has also been shown that overexpression of *Rpl23* results in resistance to T cell-mediated killing in melanoma (146). *Rps19* has been shown to correlate with the production of immunosuppressive cytokines including TGFβ, and with the inhibited infiltration of CD8^+^ T cells in breast and ovarian tumors (147). However, neither of these examples assess whether these are ribosome-associated functions and if the ribosomal incorporation of these RPs is changed nor whether these are a result of extra-ribosomal functions.

Although it is not cancer related, a recent study has shown that bacterial infection of *Arabidopsis thaliana* resulted in an altered phosphorylation state of the P-stalk proteins, which coincided with their dissociation from actively translating ribosomes. Further, plants deficient in P-stalk components showed decreased immune function, further underlining their important role in immune response (148). The authors hypothesized that the P-stalk proteins actively regulate the translation machinery to allow efficient immune defense. This is in line with other recent studies showing that P-stalk proteins are needed to translate viral mRNAs encoding transmembrane-domains (TMDs) (149). The preferential translation of TMD-containing mRNAs would therefore disproportionately affect members of the APP machinery, such as MHC or TAP, further highlighting the potential of P-stalk containing ribosomes to regulate antigen presentation and immune responses in particular.

## Therapeutic approaches and resistance

Ribosomes perform a crucial biological function, and targeting them is challenging. Until now, most therapeutic strategies have focused on targeting protein synthesis in general, and lack stratified treatment approaches. For example, increased ribosome biogenesis and high protein synthesis levels have been exploited for general targeting in cancer, using antibiotics (150–152). Although valuable, this approach would likely result in toxicity that arises from targeting such unspecific and general cellular processes. Other therapies aiming for a more specific impact on translation have used inhibitors of initiation factors, such as eIF4A or eIF2ɑ, or mTOR, hindering ribosome biogenesis and translation initiation (150), which once again, are not very specific cancer targets. The emerging field of ribosome heterogeneity and specialization therefore brings a new possibility for exploring translation machinery differences and their consequences in translation regulation between cancer and healthy cells to efficiently and specifically eliminate tumors with less impact on healthy tissues (150,151).

Patient stratification based on ribosome heterogeneity could be applied to tailored therapeutic strategies. RP mutations could be of particular interest in this context, and it was shown that tumor cells harboring the T-ALL associated mutation *Rpl10* R98S are highly sensitive to clinically used Jak-Stat inhibitors, due to high activation of this pathway promoted by uL16 R98S-containing ribosomes (as mentioned in previous sections) (43). Until now, this is the only case in which RP-mediated ribosome heterogeneity was explored for cancer treatment, but with the advent of new findings in the field of ribosome specialization, we can expect that new therapeutic approaches centered on the ‘onco-ribosome’ will emerge.

Besides RP mutations, ribosomal RP content has been shown to impact therapy resistance. One example is the above-mentioned role of RACK1-containing ribosomes in promoting translation of survival genes, which results in chemotherapy resistance in vitro (50). Ribosomal RACK1 has also been described to bridge two types of stress responses: promotion of stress granule (SG) formation by stresses such as hypoxia or arsenite exposure, and activation of the stress-activated MAPK (SAPK) pathways, known to happen upon exposure to X-rays or genotoxic drugs. 40S-associated RACK1 is incorporated in SG and this leads to a decrease in RACK1/MTK1-mediated activation of the SAPK signaling cascade, ultimately leading to apoptosis evasion. This ribosomal RACK1 role could be behind the mechanism of hypoxia-induced resistance to chemotherapy (153).

Finally, phosphorylated eS6 has been explored as a major target of interest in the past years, due to its role as a proxy for mTORC1 pathway activation, and increased levels of phosphorylated eS6 are common in cancer (35). While direct targeting of RPs is challenging, the availability of protein kinase inhibitors to treat various cancers has increased enormously over the past years (154). This could be particularly relevant to target kinases known to phosphorylate specific RPs, such as eS6 kinases for example. Indeed, eS6 kinases are currently explored as promising cancer targets (155), underlining the great potential of ribosome heterogeneity for development of new treatment strategies in cancer.

## Technical challenges

While the field of ribosome specialization has expanded significantly over the last number of years, it must be acknowledged that challenges still exist in both the methodologies used, and in the interpretation of the resultant data.

### Detection

In general, most studies have used mass spectrometry (MS) approaches to probe the existence of different ribosome subpopulations. Although MS has provided substantial information on RPs that are potentially heterogeneous in the ribosome (3), this technology poses several challenges. RPs are generally small proteins which do not possess many tryptic digestion sites (156–158), rendering them difficult to accurately and consistently detect. Additional experimental (159,160) and bioinformatic approaches are being developed (161), which should overcome some of these issues. Structural analysis using cryo electron microscopy or cryo electron tomography hold much promise in this regard, potentially allowing the direct *in situ* visualization of ribosome populations (159). Regardless of the methodology employed, confirmation of RP incorporation into the ribosome by an orthogonal method is crucial. Fluorescently labeled RPs have been used (53), but these are unlikely to be suitable in the majority of cases due to both the highly structured nature of the ribosome, and the abundance of RPs. The most common approach is still western blotting, but this can still prove challenging, particularly for samples with limited material.

### RP knock-down

The most broadly used approach to functionally assess RPs is the knock-down of the target protein. However, this poses many challenges. Ribosomes are obviously essential, so complete knock-down often affects ribosome biogenesis and has deleterious consequences for cells. As a result, the outcomes observed can strongly depend on the levels of RP knock-down, which is an obvious complication. Both inducible systems and degron technologies may be better suited to study RPs, although both present their own drawbacks. Regardless of the methodology however, it is essential to evaluate the impact on overall translation, and to control for any changes. As we have detailed above, changes in the concentration of ribosomes in a cell can have distinct phenotypes (13), and changes in RP levels are known to result in nucleolar stress (162).

Another major issue with knock-down approaches is that RPs have well described roles outside of the ribosome (163). It is just as plausible that phenotypes observed following RP knock-down are as a result of these, rather than any change in ribosome sub-populations. More targeted genetic manipulations (such as mutation of rRNA binding sites or phospho-sites) may help overcome this problem, but the direct assessment of ribosome function using a tagged RP approach results in the clearest indication of a ribosomal function. The use of proper controls, such as unaltered counterparts as well as the depletion of other RPs, is obviously key.

### Tagged RPs

As we have emphasized throughout this review, the detection of heterogeneous ribosomes is not in itself evidence for specialized ribosomes. The gold standard in this regard is the expression of tagged RPs, which can be used to isolate ribosomes that have incorporated that RP. This material can then be analyzed via ribosome profiling to identify differences in mRNAs translation between ribosome populations. While the results of such an experiment are very convincing, there are several caveats that must be taken into account when analyzing such data. First, adding a tag to an RP may change its function or ribosome incorporation. C-terminal tagged eL15, for example, cannot be incorporated during ribosome biogenesis (164), and the HA-tagging of uS14 introduces sensitivity to Ltv1 deletion in yeast, something that does not happen with untagged uS14 (165). These examples highlight that it is crucial to ensure that any tag added to an RP does not affect ribosome biogenesis, overall translation rate, or the mRNAs being translated by these ribosomes.

In most cases, the tagged RP is over-expressed, which can also result in nucleolar stress (162,166). The resulting activation of p53 drastically changes the cellular phenotype, confounding the analysis of the resulting data, so it is highly recommended that p53 activation is tested following expression of a tagged RP. An alternative to over-expression is the tagging of the endogenous locus (167), which is technically more difficult, but ensures that RP levels are not significantly altered, and at the same time overcomes any potential competition between the tagged version and the endogenous allele. More sophisticated approaches, such as cell cycle-specific tagging approaches (168), have also been developed, and the use of such approaches should allow for more nuanced measurement of translation. Obviously, the use of proper controls, such as the tagging of other RPs to compare between populations, is of the utmost importance, as is highlighted by Ferreti *et al* (16).

### Tumor heterogeneity

A final consideration is that of intercellular ribosome heterogeneity. The studies covered in this review have assumed homogeneity in the tumor. However, cancers are known to be extremely heterogeneous entities (169), and considering the known inter-tissue differences in ribosomes (170), it is likely that ribosomes differ in different areas of a tumor. The rise of single cell proteomic technologies will allow us to overcome this issue (171), but these are in their infancy, and have not as yet have been applied to the study of ribosomes. Understanding the interplay between these forms of heterogeneity will be key in deciphering the role of ribosome specialization in cancer, and when developing new targeted therapies based on this field.

## Discussion

Ribosome heterogeneity and specialization is an emerging field, and one with broad implications. There are more ribosomes in the cell than almost anything else, and if a macromolecular complex as abundant as the ribosome has direct regulatory activity, it presents significant implications for our understanding of gene expression. In the context of cancer, the field is in its infancy, so there are many open questions, which can broadly fit into two categories:

1. How does cancer change the ribosome? For example:
  - How is the ribosome reshaped by oncogenic processes?
  - How are these changes mediated?
  - Do changes in ribosome biogenesis (which are common in cancer) result in alterations in ribosome sub-populations?
  - Are ribosomes dynamically altered in cancer?
2. How do changes to the ribosome support cancer? For example:
  - Are specialized ribosomes sufficient to drive oncogenic processes?
  - Which oncogenic processes are supported by specialized ribosomes?
  - Do specialized ribosomes reshape the tumor microenvironment (TME), via the translation of secreted factors, matrix deposition, for example?
  - How do variations in composition alter the function of the ribosome?

This list highlights how much work there remains to do in this field. Cancer is an extraordinarily complex disease with many etiologies, and we have barely scratched the surface of the role of specialized ribosomes. It is simply not something that has been extensively studied yet, neither in cancer cells, nor the myriad of other cell types that make up the TME. The hypothesized immunoribosome, for example, could have a significant impact on T-cell activity and tumor immune surveillance via the direct regulation of the antigen repertoire of a tumor cell (145). Other cell types, such as dendritic cells (DCs) and macrophages, also require efficient antigen presentation to initiate an adaptive immune response (172), so it would be interesting to probe the role of the immunoribosome in these cells.

While we have focused on RPs in this review, ribosome heterogeneity has been described in many other flavors, including RP paralogs, rRNA sequence variations and modifications, and ribosome-associated proteins (173). However, only a handful of studies have provided direct evidence of functional ribosome specialization. This is partly due to the fact that ribosome heterogeneity is technically challenging to study, but also due to the prevailing view that the ribosome is a monolithic molecular complex, without active regulatory function. As a result, most studies have either excluded RPs from their data analysis, or have focused on extra-ribosomal roles (11). The majority have investigated RPs at the gene expression level across cancer types, while studies on RP incorporation into the ribosome are rare. This is problematic, as RP mRNA expression and protein abundance are poorly correlated (174), and the level of ribosome incorporation is not necessarily dependent on either, which demonstrates our lack of knowledge in this regard. For example, it is known that heterogeneous ribosomes can translate specific subpools of mRNAs (3,175), but we still don’t know the mechanism behind this, nor how common it is. Ultimately, this underlines the need for more sophisticated approaches to understand the role of ribosome specialization in cancer.

Throughout this review, we have highlighted the current lack of evidence for ribosome specialization, which is a direct result of the lack of techniques available to identify it. However, the field of ribosome heterogeneity and specialization is developing extremely rapidly, and our toolbox is expanding. In the coming years we expect a substantially deeper understanding of what defines ribosome specialization, and what signals drive it. This field has proven to be an exciting and surprising one. Developing an increased appreciation of the role of ribosomes in cancer cells will hopefully answer some of the many outstanding questions in the field, and may even present new opportunities in patient stratification and therapy.

## Data Availability

There are no new data associated with this article.
